# Synonymous Codons and Hydrophobicity Optimization of Post-translational Signal Peptide PelB Increase Phage Display Efficiency of DARPins

**DOI:** 10.1021/acssynbio.2c00260

**Published:** 2022-09-30

**Authors:** Antti Kulmala, Matias Lappalainen, Urpo Lamminmäki, Tuomas Huovinen

**Affiliations:** † Department of Life Technologies, University of Turku Kiinamyllynkatu 10, 20520 Turku, Finland

**Keywords:** signal sequence, DARPin, phage display, synonymous codons, periplasmic expression, protein engineering

## Abstract

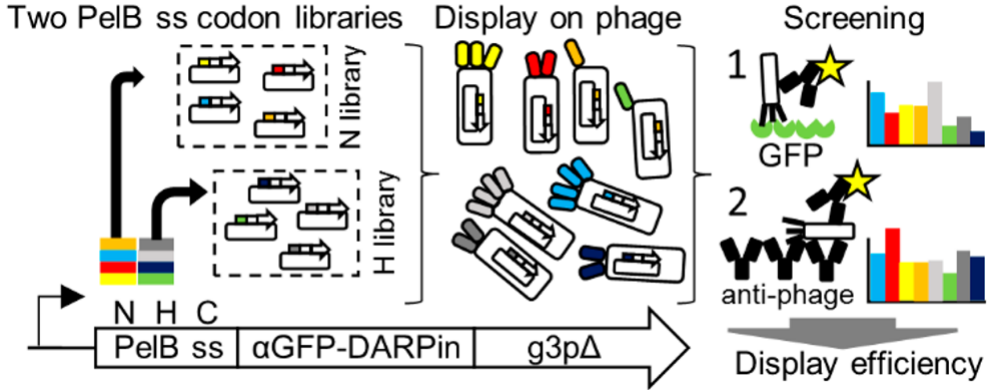

DsbA leader peptide targets proteins for cotranslational translocation by signal recognition particle (SRP) pathway and has been the standard signal sequence for filamentous phage display of fast-folding Designed Ankyrin Repeat Proteins (DARPins). In contrast, translocation of DARPins via the post-translational pathway, for example, with the commonly used PelB leader, has been reported to be highly inefficient. In this study, two PelB signal sequence libraries were screened covering different regions of the leader peptide for identifying mutants with improved display of DARPins on phage. A PelB variant with the most favorable combination of synonymous mutations in the n-region and hydrophobic substitutions in the h-region increased the display efficiency of a DARPin library 44- and 12-fold compared to PelB_WT_ and DsbA, respectively. Based on thioredoxin-1 (TrxA) export studies the triple valine mutant PelB DN5 V3 leader was capable of more efficient cotranslational translocation than PelB_WT_, but the overall display efficiency improvement over DsbA suggests that besides increased cotranslational translocation other factors contribute to the observed enhancement in DARPin display efficiency.

Designed Ankyrin Repeat Proteins (DARPins) are highly expressing, monomeric, and stable artificial binder molecules,^1^ which are used in many different application areas such as basic biochemical research,^2−4^ diagnostics,^5^ and therapy.^6−8^ Ribosome display has been the preferred method for the evolution of DARPins against various targets,^9−12^ because of very large attainable library sizes and compatibility of DARPins with cell-free translation.^13,14^ However, in some applications requiring a robust display platform, e.g., for biopanning on whole cells or working with challenging pH and temperature conditions, phage display would be better suited.^15,16^ Since DARPins fold very fast and efficiently in the cytoplasm,^17^ phage display of DARPins has been reported to be highly inefficient with the most commonly used post-translational Sec pathway^18^ in which SecB guides proteins in an unfolded state to the SecYEG translocon.^19^ Steiner and colleagues discovered that the display of DARPins can be enhanced remarkably by using a cotranslational SRP (signal recognition particle) pathway instead of a post-translational pathway.^18^ In general, however, the transportation capacity of the post-translational pathway is greater than the transportation capacity of the SRP pathway, and the SRP pathway is more prone to overloading, which in turn can induce toxic effects on the host cell.^20−22^ Therefore, the improvement of the post-translational translocation of DARPins would be an attractive upgrade for the DARPin phage display technology. It has been discovered that codon usage of signal sequences plays a major role in the expression and translocation of different proteins.^23−26^ Recently, we enhanced the expression and Sec-dependent translocation of a Fab fragment by selecting improved variants from PelB signal sequence libraries,^24^ which included synonymous codon alterations in the n-terminal (n-region), hydrophobic region (h-region), or c-terminal region of the PelB signal sequence originating from the pectate lyase B gene of *Erwinia carotovora*.^27^ The results gained with Fabs inspired us to explore the possibility of enhancing the phage display of DARPins similarly by using synonymous PelB signal sequence libraries.

## Results and Discussion

**Figure 1:**
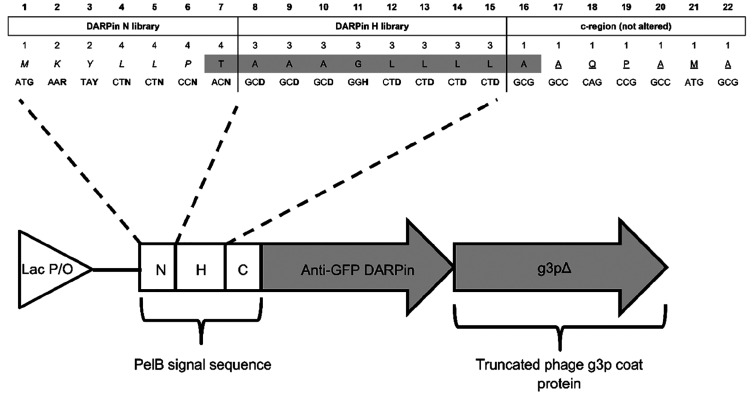
The design of the synonymous codon libraries DARPin N and DARPin H. N library contained synonymous codon variation in PelB residue positions 1–7 and H library in positions 8–15, respectively. In order to balance the number of combinatorial variants in the N and H libraries, the H library covered a two-codons shorter region than typically defined as the hydrophobic region (gray shading), and the N library covered one-codon more than defined as the n-region (letters in italics). The c-region was not randomized (underlined letters). Phage display experiments were carried out by fusing the libraries to an anti-GFP DARPin and a C-terminal g3pΔ phage capsid gene (i.e., p3-CT, lacking N1 and N2 domains). The expression of DARPin-g3pΔ fusion proteins was controlled with Lac promoter (LacP/O). The number of synonymous codon variants at each position is shown above the amino acid sequence of the PelB signal sequence. **D** = A, G, T; **Y** = C, T; **H** = C, T, A; **V** = A, G, C; **R** = A, G.

DARPin N and DARPin H libraries carrying synonymous codon mutations in the n-region or h-region of the PelB signal sequence (Figure 1), respectively, were screened for improved anti-GFP DARPin display by picking 900 clones from both libraries and producing phage in the wells of 96-well plates. Subsequently, the produced phage was analyzed by two immunoassays, one of which measured the specific binding of phage to biotinylated GFP, and the other that measured the total amount of phage in the sample. The ratio of target binding signal to total phage mass was used to compare display levels between different clones. Clones showing higher DARPin display level than DsbA, as assessed by single-point primary screening of DARPin N and DARPin H libraries, were selected for secondary screening, in which phage production, immunoassays, and analysis were performed as five independent replicates.

The results of the secondary screening with DNA sequences are presented in Table 1. In this screening, seven N library members displayed anti-GFP DARPin on phage at a higher level than PelB_WT_ (Mann–Whitney U-test, *p* < 0.05), out of which, the best variant DN5 enabled 9-fold (*p* = 0.009) and 2-fold (*p* = 0.027) higher display level than PelB_WT_ and DsbA, respectively. In addition to synonymous mutations, one A8V substitution was found in the DN5 sequence. Among the DARPin H library members, variants DH4 and DH7 displayed anti-GFP DARPin with 10-fold (*p* = 0.009) and 5-fold (*p* = 0.009) higher efficiency than PelB_WT_, but no statistically significant improvement was observed as compared to DsbA (Table 1).

**Table 1:**
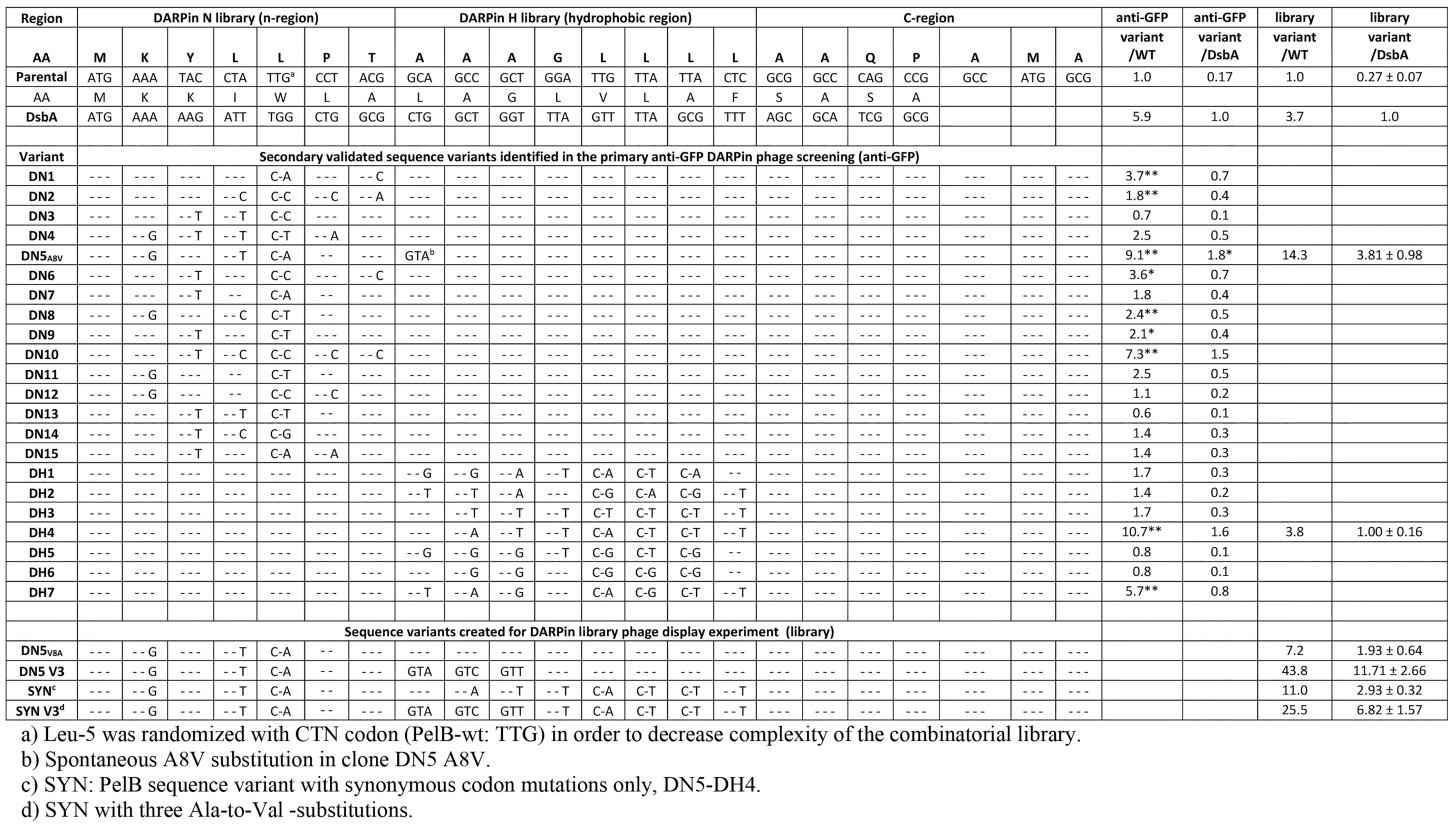
Summary of DARPin Phage Display Improving Mutations in Anti-GFP and Binary Ser/Tyr Library Display Experiments

**Figure 2:**
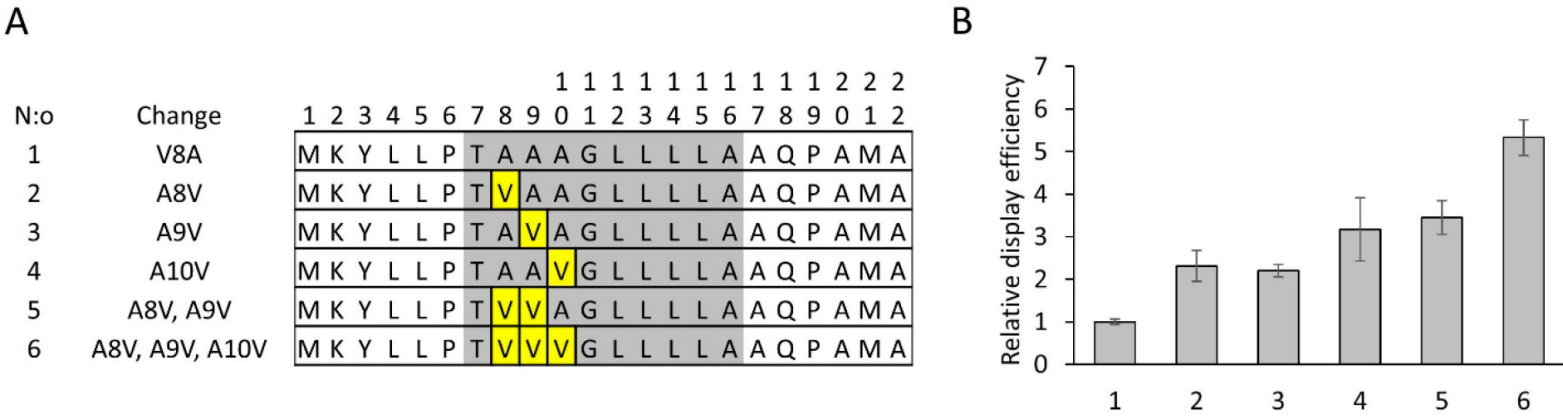
Effect of hydrophobicity engineering of variant DN5 PelB signal sequence h-region on average display level of the anti-GFP DARPin on phage. (A) Summary of tested DN5 mutants with substitutions in the h-region. (B) Phage display efficiency of DN5 mutants. The phage display efficiency is shown as relative to the DN5 V8A variant that does not have any valine substitutions. Error bars represent standard deviation of five independent series of phage productions.

The significance of the hydrophobic substitution A8V located in the h-region of variant DN5 on display efficiency was analyzed by constructing the V8A reverse mutant. In addition, further variants containing either one (A9V or A10V), two (A8V and A9V), or three (A8V, A9V, and A10V, renamed V3) Ala-to-Val substitutions were created for analyzing possible positional bias and additive effects. The analysis with anti-GFP DARPin phage confirmed that the display efficiency correlated with the number of valine substitutions (Figure 2). Single substitution in either position 8 or 9 doubled the display efficiency, whereas a substitution at position 10 tripled the efficiency indicating a small positional effect. Highest DARPin display on phage (5-fold compared to the DN5 V8A variant) was achieved with all three Ala-to-Val substitutions.

Next, we investigated the transferability of the observed display improvement to other DARPins by fusing leader peptide variants to a DARPin library gene pool containing 23 residue positions with combinatorially varying serine/tyrosine amino acids (termed binary library with TMY codons at the selected paratope positions, see Supporting Information for details). In order to analyze whether synonymous mutations in the n- and h-regions would have additive effects on display efficiency, a double mutant DN5–DH4 was created that did not contain A8V substitution. Yet another sequence variant was prepared containing both synonymous mutations (as in DN5-DH4) and the three beneficial Ala-to-Val substitutions. In this experiment PEG/NaCl-precipitated phage stocks were used as the sample material instead of growth medium directly as it simulates better the procedure in biopanning selections. Furthermore, phage propagation protocol was simplified. Previously, growth medium was changed from high-glucose to low-glucose after phage superinfection (to avoid premature DARPin expression), and phage were allowed to propagate overnight at 37 °C (see Figure S2). In this experiment, no glucose was added to the medium, and the cultures were propagated overnight at 30 °C after superinfection. After phage stock preparation, DARPin-phage particles (5 × 10^9^ pfu per well) were captured on microtiter plate wells with anti-DARPin Fab fragments and detected with europium labeled antiphage antibody.

**Figure 3:**
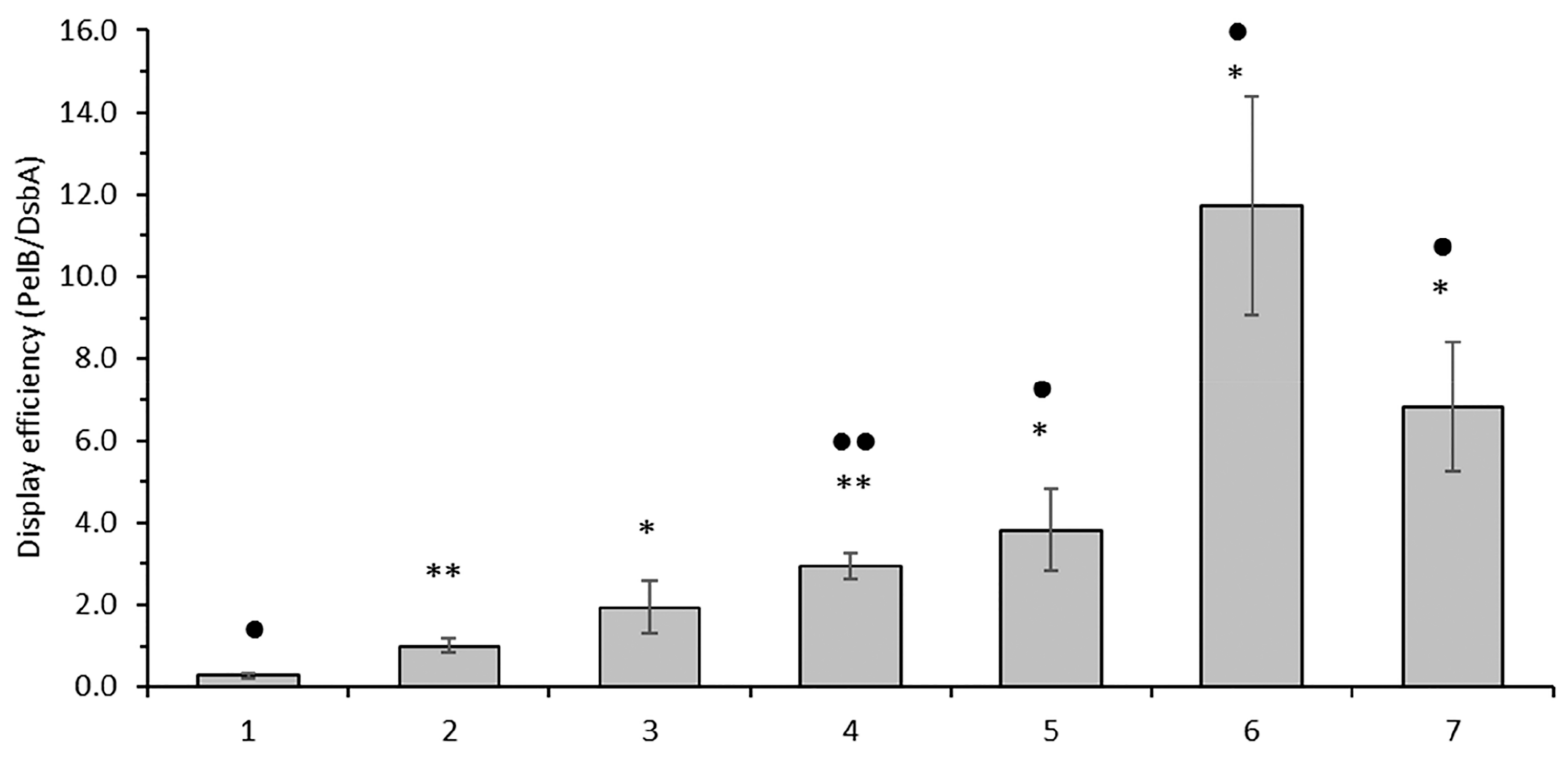
Effect of synonymous codons and valine-substitutions in PelB signal sequence on average phage display levels of DARPin serine/tyrosine binary library. (1) PelB wt, (2) DH4, (3) DN5 V8A, (4) DN5-DH4, (5) DN5 A8V, (6) DN5 V3 and (7) DN5-DH4 A[8,9,10]V. The DARPin display levels are shown as relative to the library samples displayed with DsbA signal sequence. Error bars represent standard deviation of three independent series of phage productions. Significant differences to the parental PelB and DsbA are shown by asterisk (∗) and sphere (●), respectively. Mann–Whitney U, *(●) *p* < 0.05; **(●●) *p* < 0.01.

In general, all tested PelB signal sequence clones exhibited statistically significant improvement in display of the binary library compared to the parental PelB (Figure 3). The mutant DN5–DH4, containing synonymous codon mutations only, displayed the binary library with 3-fold higher efficiency than DsbA. Interestingly, the display efficiency was only doubled by complementing the most optimal synonymous codon variant further with the three Ala-to-Val substitutions (Figure 3, clone 7), whereas the display efficiency was quadrupled by complementing the variant DN5, that contained synonymous codon mutations only in the n-region, with the triple valine mutations. It is worth noting that the synonymous codons of the h-region of DH4 variant could be only partially transferred to the variant DN5-DH4 A[8,9,10]V as the three substitutions are, *ipso facto*, overlapping with the h-region (Table 1). Changes in phage production protocol had a minor effect on display efficiency, the latter protocol (without glucose supplementation and with 30 °C propagation overnight) being superior to the earlier protocol with medium change from high-to-low glucose and propagation overnight at 37 °C (see Figure S2 for comparison). The most efficient leader peptide DN5 V3 displayed the binary DARPin library with 12-fold higher efficiency than DsbA (PelB_DN5 V3_/ DsbA: 11.7 ± 2.7).

The mechanism for the improved phage display efficiency promoted by the PelB variants was further studied by constructing PelB-thioredoxin-1 reporter constructs (see supplementary for construct details). Thioredoxin-1 (TrxA) is a cytoplasmic rapidly folding protein of *E. coli* that according to Huber et al. (2005) transports to the periplasmic space only via cotranslational translocation and, therefore, serves as a reporter protein for analyzing signal peptides capable of cotranslational translocation.^28^ Periplasmic extracts of PelB-TrxA fusions were prepared by a similar method as described by Huber et al. (2005) and analyzed by Western blotting with an anti-His tag HRP antibody.

**Figure 4:**
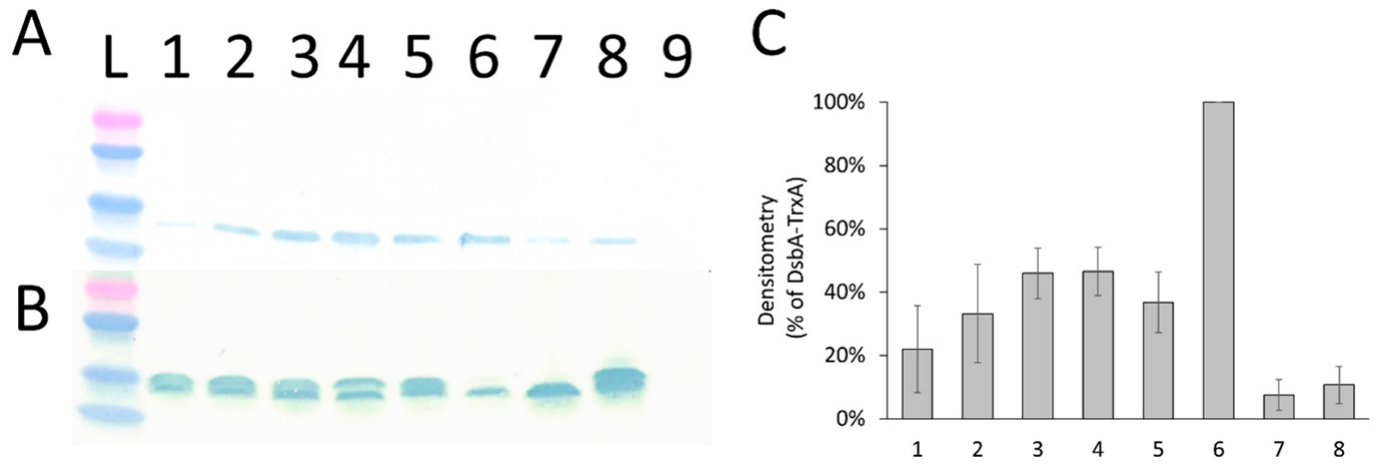
Western blot analysis of thioredoxin-1 (TrxA) reporter assay. (A) Samples of periplasmic fractions. (B) Samples of whole cells. (C) Western blot densitometry of five independent series of periplasmic extractions. The band intensities of PelB-TrxA variants and controls were normalized to the total protein expression obtained by Western blotting whole cell samples and compared to the positive control DsbA-TrxA (6). Samples of periplasmic extracts and whole cells were run on 4–15% SDS-PAGE, transferred on PVDF and probed with anti-His tag HRP. Precipitating TMB substrate was used for the detection. (L) Ladder: 5 μL Precision Plus Protein Standard Dual Color (BioRad, USA); (1) PelB_WT_; (2) PelB DN5 V8A; (3) PelB DN5 A8V; (4) PelB DN5 V3; (5) DN5-DH4; (6) DsbA; (7) TrxA without signal sequence; (8) PhoA; (9) XL-1 ctrl.

The Western blot analysis confirmed the findings reported earlier that DsbA leader peptide directed the TrxA efficiently to the periplasm and that the archetypical post-translational translocation signal peptide PhoA inefficiently (Figure 4A). However, a faint band was seen both in the PhoA-TrxA sample as well as in another negative control containing TrxA without a signal sequence. Despite of the narrow quantitativeness of the reporter assay, it can be observed that the hydrophobicity-engineered leaders (DN5 A8V and DN5 V3) export TrxA to the periplasm more efficiently than the synonymous codon variants (Figure 4C). Because DN5 V3 outperformed DsbA 12-fold in the phage display efficiency assay, but had only half of the potency of DsbA in the TrxA reporter assay, the increased periplasmic export of DARPin-g3pΔproteins in phage display experiments cannot be explained by the improved cotranslational translocation efficacy alone. Furthermore, DsbA-TrxA was fully cleaved (band corresponding to the cytoplasmic TrxA without signal sequence), whereas PelB-TrxA variants are present with and without the signal sequence in the whole cell samples.

**Figure 5:**
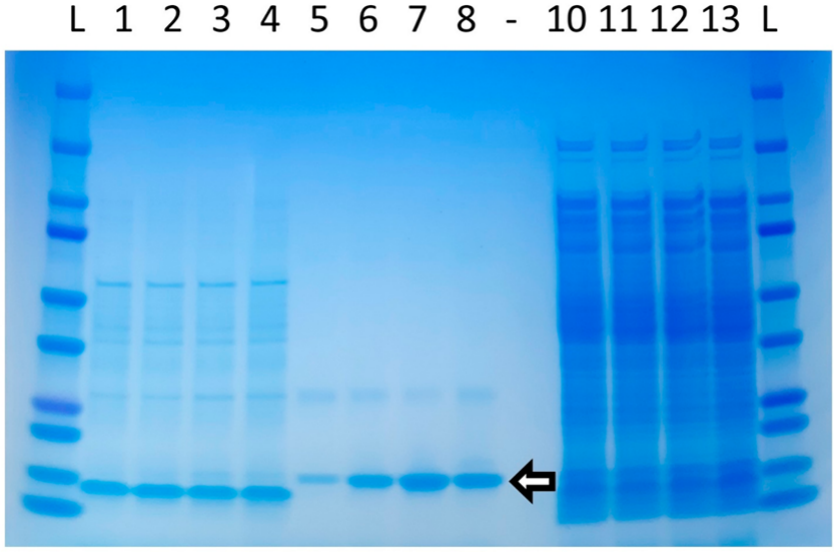
Periplasmic extraction of soluble anti-GFP DARPin translocated with novel PelB signal sequence variants. Periplasmic fraction samples before NiNTA, lanes 1–4: (1) PelB wt, (2) ND5, (3) ND5 V3, and (4) ND5-DH4. Periplasmic fractions samples after NiNTA elution, lanes 5–8: (5) PelB wt, (6) ND5, (7) ND5 V3, and (8) ND5-DH4. Cytoplasmic fraction prepared by 3x freeze–thawing the remaining pellets from periplasmic extraction, lanes 10–13: (10) PelB wt, (11) ND5, (12) ND5 V3, and (13) ND5-DH4. Precision Plus Protein Dual Color Standard (BioRad, USA) was used as the protein size marker (L) and the white arrow on the empty lane 9 indicates the DARPin (18.0 kDa). The thick band below the DARPin band in the periplasmic extraction samples (also visible in cytoplasmic fraction) is egg hen lysozyme (14.3 kDa) used for periprep extraction.

Then, we set out to study whether the leader DN5-DH4 (with synonymous codons only), DN5 A8V, and DN5 V3 (with three valine substitutions) have a beneficial effect on soluble periplasmic expression of DARPins and if the signal sequences are cleaved off from the DARPin by the signal peptidase. The periplasmic fraction was prepared with the TES-lysozyme protocol, and DARPins were purified with NiNTA-chromatography (C-terminal His tag) prior to analysis with LC–MS. Based on SDS-PAGE analysis, DN5 V3 expressed with the highest efficiency in the periplasm (Figure 5). The mass spectrometry analysis confirmed efficient cleavage of the signal sequence as the most intense peak in all periplasmic samples represents an average mass of 18 045 Da (see Figures S4 and S5), which matches well with the theoretical mass of anti-GFP DARPin without the secretion signal (18 045.28 Da). The other abundant peaks detected in all samples represent average masses of 18 173 Da and 20 846 Da, the latter of which, is slightly larger than the mass of uncleaved PelB-DARPin protein (theoretical masses for WT/ND5-DH4-, ND5-A8V-, and ND5-V3-PelB DARPins are 20 256 Da, 20 284 Da, and 20 340 Da, respectively).

In this study, PelB signal sequence libraries containing synonymous codon mutations in the n-region, or hydrophobic region of the PelB signal sequence^24^ were screened through for higher phage display efficiency of DARPins. Synonymous codon variants, e.g., ND5-DH4, that displayed DARPins 3-fold more efficiently on phage than DsbA, the golden standard leader peptide for SRP-dependent phage display, were identified. Furthermore, due to the serendipitous discovery of A8V substitution in the h-region, a series of h-region variants were constructed containing an increasing hydrophobicity which correlated with higher display efficiency. The highest performing mutant, signal peptide DN5 V3, had a combination of synonymous mutations in the n-region and hydrophobicity increasing substitutions in the h-region leading to 12-fold improved DARPin phage display efficiency as compared to DsbA.

Increasing hydrophobicity of the h-region has been linked to transition from post- to cotranslational translocation and improved cotranslational secretion.^28−30^ Our findings support this conclusion as the identified hydrophobicity increasing h-region mutations promoted periplasmic expression of TrxA which is considered an archetypical reporter protein exploiting the SRP-dependent cotranslational translocation route. Recent studies suggest that cotranslational translocation is more common in *E. coli* than earlier considered. For example, SecA is able to bind to nascently synthesized peptides on ribosome and engage the complexes to SecYEG translocon for coupled translation.^31^ New results suggest a complex export process for G3p that makes classification into post- or cotranslational translocation ambiguous as both SRP and SecB targeting systems could be mutually functional for translocation of the N-terminal infection domains N1 and N2 to the periplasm followed by membrane insertase YidC mediated integration of the C-terminal anchor domain to the inner membrane.^32^ In our study the native signal sequence and infection domains of G3p were replaced with the modified signal sequence and DARPin followed by the C-terminal domain. Detailed elucidation of the export route and targeting partners of the DARPin fusion protein would require further studies, but based on data from the wt G3p export process, phage display, and TrxA reporter experiments, it is speculated that the improved DARPin display with PelB DN5 V3 is a result of using both post- and co-translational export routes more efficiently.

In conclusion, synonymous codon libraries and hydrophobicity engineering are powerful tools for achieving successful phage display of cytoplasmic proteins, expanding the armada of proteins amenable to combinatorial protein engineering approaches.

## Materials and Methods

### Cloning of Anti-GFP DARPin Gene into Synonymous PelB Signal Sequence Libraries

Primer sequences used in this study are given in Table S1. All the primers were obtained from Sigma-Aldrich (St. Louis, USA) and all the enzymes were obtained from Thermo Scientific. The anti-GFP DARPin gene 3G86.32^33^ was cloned into two previously described synonymous PelB signal sequence libraries^24^ of which one contained synonymous codon alterations in the n-region and another in the hydrophobic region of PelB signal sequence present in vector pEB32x using SfiI sites and T4 DNA ligase with standard molecular cloning procedures. The library DNA was transformed to *E. coli* SS320 (MC1061 F’) cells,^34^ and the cells were plated on LA (0.5% glucose, 25 μg/mL cm, 10 μg/mL tet) after recovery. After overnight incubation at 30 °C, the cells were scraped from the plates and phage stocks were produced from both libraries as described by Kulmala et al.^35^ The only exception was the overnight temperature for phage propagation, which in this case was 37 °C. The phage titers were determined by oligonucleotide-directed chelate complementation assay (OCCA)^36^ in the same manner as previously described by Kulmala et al.^35^ Details of the library preparation are provided in the supplementary methods.

### Screening of the DARPin N and DARPin H Libraries

The *E. coli* XL1-Blue cells were infected with DARPin N and DARPin H library phages in two separate infection reactions with 20-fold multiplicity of infection at 37 °C for 40 min without shaking. In addition, XL1-Blue cells were infected with phages harboring DARPins equipped with parental PelB signal sequence or DsbA signal sequences, and these signal sequences were used as controls throughout the study. The infection reactions were diluted and plated on big LA plates (0.5% glucose, 25 μg/mL cm, 10 μg/mL tet). The next day, 900 colonies from each library plating, DARPin N and H, were picked to the wells of 96-well microtiter plates containing 160 μL of SB medium per well (1% glucose, 25 μg/mL cm, 10 μg/mL tet). Subsequently the plates were covered with breathable tape (Thermo Scientific) and incubated overnight at 37 °C with 900 rpm shaking in 70% humidity. After overnight incubation, the 96-well microtiter plates containing 200 μL of SB medium per well (1% glucose, 25 μg/mL cm, 10 μg/mL tet) were inoculated with 5 μL of overnight precultures. The plates were covered with breathable tape and incubated at 37 °C with 900 rpm shaking in 70% humidity for 3 h until the cultures looked turbid. Then, the cultures were infected with 50 μL (1.6 × 10^9^ pfu) of VCS-M13 helper phage (Stratagene, LaJolla, USA) and incubated at 37 °C for 45 min without shaking. After infection, the cells were pelleted by centrifugation (10 min, 805g, RT), and medium was discarded. The cells were resuspended in 200 μL per well of fresh SB medium (25 μg/mL cm, 10 μg/mL tet, 5 mM MgCl_2_). The plates were covered with breathable tape and incubated at 37 °C with 900 rpm shaking in 70% humidity overnight. After overnight culture, the cells were pelleted by centrifugation (15 min, 3220g, + 4 °C), and supernatants containing phages were collected.

Two different immunoassay setups were established for the detection of the phages. The first one was based on specific binding of the phage displaying anti-GFP DARPin to biotinylated GFP. The second one was based on biotinylated mouse antiphage antibody (University of Turku, Turku, Finland), which binds to the abundant pVIII coat protein of the phage, thereby measuring the total phage amount. First, wells of a 96 well streptavidin plate (Kaivogen, Turku, Finland) were coated with 30 pmol of biotinylated GFP in 100 μL of Assay Buffer (Kaivogen) or with 25 ng of biotinylated mouse antiphage antibody in 100 μL of Assay Buffer. The plates were incubated at RT for 30 min with slow shaking and subsequently washed two times with Delfia plate wash (Wallac, Turku, Finland) by using Kaivogen Wash Buffer. Next, 10 μL (specific phage assay) and 1 μL (total phage assay) of the phage stocks initially diluted 1/100 in Assay Buffer were pipetted to the wells, and subsequently Assay Buffer was added to the final volume of 100 μL per well. The plates were incubated at RT for 30 min with slow shaking and subsequently washed two times. Bound phages were detected with 25 ng per 100 μL Eu-labeled mouse antiphage antibody (University of Turku, Turku, Finland) in both assays. The plates were incubated at RT for 30 min with slow shaking and subsequently washed four times. Next, 200 μL of Enhancement solution (Wallac) was added, and the plates were incubated at RT for 10 min with slow shaking. After incubation, the time-resolved europium signal was measured with a Victor 1420 Multilabel Counter (Wallac).

### DARPin Library Display Assay

Construction of the DsbA leader library and DN5–DH4 PelB sequence variant is described in the supplementary methods. The DARPin binary library was purchased as a ready linear DNA block from Eurofins Genomics (Ebersberg, Germany) containing a serine/tyrosine codon variation (TMY) at specified codon positions (Figure S1). The library was amplified with PCR using added flanking primer sites and digested with SfiI. All PelB variants present in pEB32x vector backbones (upstream from the first SfiI site) were ligated to the DARPin binary library and transformed to *E. coli* XL-1 Blue cells with electroporation, each yielding >10 000 cfu transformants. All PelB mutant libraries contained <0.7% vector background colonies, which were calculated from the transformation plates of vector control ligations (no insert) that were prepared in parallel to the library samples.

Overnight precultures were diluted to OD600 0.05 in 20 mL of SB medium (25 μg/mL cm, 10 μg/mL tet), and cells were cultured at 37 °C with 300 rpm shaking to OD600 0.5. Cells were infected with VCS-M13 helper phage (Stratagene) with 20-fold multiplicity of infection and incubated at 37 °C for 30 min. Cultures were grown at 30 °C for 1 h with 250 rpm shaking, after which kanamycin was added to a 30 μg/mL final concentration and incubation continued on. The following day, cultures were centrifuged at 6800*g* for 10 min at 4 °C. Supernatants were collected, and 1/6 volume of 20% PEG-8000/2.5 M NaCl was added to precipitate the phages. Precipitation reactions were incubated on ice for 1 h and subsequently centrifuged at 6800*g* for 15 min at 4 °C. Phage pellets were dissolved in 1 ml of TSA buffer (50 mM Tris-HCl, pH 7.5; 150 mM NaCl, 0.02% w/v NaN_3_) and subsequently centrifuged with a tabletop centrifuge at 16 300*g*, for 5 min at 4 °C to remove remaining cells. Supernatants were transferred to new tubes. The phage productions were repeated three times on different days.

The capability of each signal sequence to display serine/tyrosine binary library DARPins on the surface of phage particles was determined by immunoassay. Wells of a 96 well streptavidin plate were coated with 100 μL per well of 250 ng/μL biotinylated 2A11 antihuman Fab (Hytest Ltd., Turku, Finland) in Assay Buffer. The plate was incubated at RT for 30 min with slow shaking. After incubation, the plate was washed two times with Delfia Plate Wash by using Kaivogen wash buffer. After the plate was washed, 100 μL per well of 600 ng/μL anti-DARPin Fab clone 245E6 (University of Turku, Turku, Finland) was added to the wells. The incubation and washing steps were done similar to that in the previous steps. Subsequently, the same amount of phage from each library phage production (5 × 10^9^ pfu) was added to the wells in 100 μL of Assay Buffer as triplicate. Again, incubation and wash steps were done as in previous steps. Bound phages were detected with 25 ng per 100 μL europium labeled mouse antiphage antibody. The incubation time was the same as above, but this time after incubation, the plate was washed four times. Subsequently, 200 μL of Enhancement solution was added, and the plates were incubated at RT for 10 min with slow shaking. After incubation, the time-resolved europium signal was measured with Victor 1420 Multilabel Counter.

### TrxA Reporter Assay

Cloning of pAK400-TrxA constructs (for sequence, see Figure S3) for the TrxA reporter assay and DARPin periplasmic export constructs for mass spectrometry are described in supplementary methods. Overnight cultures of XL-1 Blue cells harboring pAK400-TrxA vectors with different signal sequences (see supplementary file for sequences) were inoculated to 10 mL of SB (1% glucose, 25 μg/mL cm, 10 μg/mL tet) to OD600 0.05. The cells were incubated at 37 °C with 250 rpm until OD600 0.5 and centrifuged at 3200*g* for 15 min at 24 °C. The cell pellets were resuspended in 20 mL of SB (100 μM IPTG, 25 μg/mL cm, 10 μg/mL tet), and incubation continued at 37 °C with 250 rpm for 4 h. The cell concentrations were normalized by diluting more turbid cultures to the same OD600 value as the less turbid cultures with SB medium. For periplasmic extraction, 10 mL of cell suspension was taken and centrifuged at 3200*g* for 15 min at 4 °C, the supernatant was decanted, and the pellets were resuspended into 500 μL of periprep buffer containing 0.5 μg/mL lysozyme (Sigma-Aldrich, USA), 18% w/v sucrose, 1 mM CaCl_2_, and 0.5 mM EDTA pH 8.0 in 50 mM Tris-Cl pH 8.0. The samples were incubated on ice for 30 min and centrifuged at 3500*g* in a tabletop microcentrifuge for 10 min at 4 °C. The supernatant, i.e., the periplasmic fraction, was assayed for TrxA with Western blotting.

For SDS-PAGE, 5 μL of periplasmic fractions were mixed with 5 μL of reduced Laemmli buffer (2x concentrate, Bio-Rad, USA) and heated for 5 min at 95 °C. The samples were run on 4–15% SDS-PAGE (Bio-Rad, USA) at 200 V for 33 min and transferred with Trans-Blot Turbo semidry transfer cell (Bio-Rad, USA) on Ø 0.2 μm PVDF with settings: 2.5 A, 25 V, and 7 min transfer time. The PVDF membrane was blocked with 10% fat-free milk (blotting grade blocker, BioRad, USA) in PBST0.05 (PBS + 0.05% Tween-20) for 30 min at RT with rocking. The membrane was incubated with 1/2500 dilution of anti-His tag HRP antibody (HRP-66005, Proteintech Group, USA) in 10% fat-free milk in PBST0.05 for 45 min. PVDF membrane was washed 3x 5 min with PBST0.05, and TrxA was detected with precipitating TMB (Sigma-Aldrich, USA).

The Western blots were quantified with densitometry by converting the images to gray scale, defining lanes with the rectangular selection tool, and creating profile plots of the lanes with the gel analysis suite of the Fiji software package (ImageJ 1.53c, W. Rasband, NIH, USA). The peak area values corresponding to the band intensity were analyzed as a percentage of the peak area of DsbA-TrxA. The periplasmic extraction yields were normalized to total expressed protein by expressing three more series of TrxA samples and taking 1 mL of cells normalized to the same optical density as above. The samples were pelleted for 5 min at 16500*g* at 4 °C, the supernatant was removed, and the pellet was resuspended in 1 mL of H_2_O. The samples were Western blotted from 1/10 dilution, and the peak intensities were quantitated with Fiji relative to DsbA-TrxA as above. The periplasmic TrxA peak intensities were divided by the mean of the total expressed TrxA protein for each sample. An example of defined lanes and peak profiles in Fiji is shown in Figure S6.

The periplasmic DARPin expression was carried out similar to the TrxA expressions, except in 500 mL volume at 30 °C with 250 rpm shaking for 6 h. The periplasmic fractions were prepared as in previous steps with the exceptions that the cell pellets were resuspended in 50 mL of periprep buffer containing increased lysozyme concentration of 0.5 mg/mL and incubated for 60 min on ice. The periplasmic fractions were purified with Ni-NTA (Thermo Scientific, USA). The periplasmic sample buffer was adjusted for IMAC by adding 5 mL of 10x PBS and 500 μL of 1 M imidazole (final 11 mM imidazole), mixed with 800 μL of prewashed Ni-NTA slurry (equivalent to 400 μL packed bed), incubated 10 min at RT in rotation, and spun at 700*g* for 3 min at RT to pellet the NiNTA matrix. The supernatant was removed, and the Ni-NTA matrix was resuspended in 4 mL of binding buffer (10 mM imidazole in PBS pH 7.4). The matrix was loaded on an empty cartridge, settled by gravity, washed twice with 4 mL of washing buffer (25 mM imidazole in PBS), and eluted with 1.2 mL of 500 mM imidazole in PBS on an ultrafiltration device (VIVASPIN Turbo 4, 10 kDa molecular weight cut-off, Sartorius, USA). The samples were concentrated, and buffer was exchanged to water (three times buffer exchange: retentate 200 μL diluted to 3.8 mL of ultrapure H_2_O) to a final volume of 500 μL. Samples of 5 μL were run on SDS PAGE (4–20% Mini-PROTEAN TGX) to verify purity.

### Statistical analysis

All the statistical analyses were performed by using IBM SPSS Statistics 22 (Armonk, USA). All the pairwise comparisons were implemented by using nonparametric Mann–Whitney U test.

## Supporting Information Available

Cloning methods, mass spectrometry run details, primer sequences, serine/tyrosine binary library design and construction, densitometry of TrxA reporter assay Western blots, and results from alternative DARPin phage production protocol (PDF)
