# Supplementary material for: Synonymous Codons and Hydrophobicity Optimization of Post-translational Signal Peptide PelB Increase Phage Display Efficiency of DARPins
Source: ACS Synth Biol. 2022 Sep 30;11(10):3174–81. doi: 10.1021/acssynbio.2c00260 (PMC9594773; doi:10.1021/acssynbio.2c00260)
Supplement: Supplementary file 1 — sb2c00260_si_001.pdf [file sb2c00260_si_001.pdf]

Supporting Information

Synonymous Codons and Hydrophobicity Optimization of Post-translational Signal Peptide PelB Increase Phage Display Efficiency of DARPins

Antti Kulmala[a], Matias Lappalainen[a], Urpo Lamminmäki[a] and Tuomas Huovinen[a*]

[a] Department of Biochemistry/Biotechnology, University of Turku

Address: Kiinamyllynkatu 10, 20520 Turku, Finland

*Corresponding author

To whom correspondence should be addressed:

Tuomas Huovinen

E-mail: tuohuo@utu.fi

Supplementary Methods

Cloning of anti-GFP DARPin gene into synonymous PelB signal sequence libraries

The synonymous codon libraries were initially established in pEB32x vector harboring Fab fragment gene, which contained two PelB signal sequences: light and heavy chain PelB. In the case of the Fab fragment, the n-region and the hydrophobic region were simultaneously diversified in the light chain PelB and in the heavy chain PelB. To remove the Fab fragment gene from the vector, the libraries were digested by using SfiI restriction enzyme (Thermo Scientific, Waltham, USA) in o/n reaction at 50 °C. The SfiI digestion leaves the light chain PelB attached to the vector but removes the heavy chain PelB. The next day, the libraries were further digested for 2 h by using XhoI restriction enzyme (Thermo Scientific) to reduce the Fab fragment background in the construction of the DARPin PelB signal sequence libraries. Subsequently, the digested libraries carried by the pEB32x vector were extracted from an agarose gel with GeneJet Gel Extraction kit (Thermo Scientific). SfiI digested anti-GFP DARPin gene was ligated into the synonymous n-region and hydrophobic region libraries in 1:3 vector/insert molar ratios by using T4 DNA Ligase (Thermo Scientific). The ligation reactions were incubated for 2 h at RT and inactivated for 5 min at 70 °C. Both ligation reactions, which contained ~300 ng of pEB32x vector DNA in 25 µl volume, were mixed with 280 µl of *E. coli* SS320 (MC1061 F') cells [34] and transformed into the cells with Bio-Rad Genepulser (Bio-Rad, Hercules, USA) with settings 2.5 kV, 25 µF, 200 Ω in two separate electroporation. After electroporation, the cells were recovered in 10 ml of SOC medium at 37 °C for 45 min with 100 rpm shaking. In order to determine the sizes of the two libraries, the recovered cells were diluted 10^{-1}-10^{-5} in SB medium, and 100 µl of the 10^{-3}-10^{-5} dilutions were plated on LA plates (0.5 % glucose, 25 µg/ml cm, 10 µg/ml tet). Plates were incubated at 37 °C, o/n. The rest of the cells were plated on big LA plates (0.5 % glucose, 25 µg/ml cm, 10 µg/ml tet) and incubated at 30 °C, o/n. The next day, cells were scraped off from the plates with 2 ml of SB medium (2 ml/plate). The library sizes of the DARPin N (synonymous alterations in the n-region of the PelB) and DARPin H (synonymous alterations in the hydrophobic region of the PelB) were 1.9 x 10^7 cfu and 1.3 x 10^6 cfu, respectively.

Construction of a serine/tyrosine binary library

Combined PelB signal sequence mutants of DN5 and DH4 (DN5–DH4) and DN10 and DH4 (DN10–DH4, assayed only in previous early phage display experiments, see **Figure S2**) were created by amplifying the pEB32x-DARPin PelB DN5 and DN10 vectors with PCR using common forward primer TH361 and mutant-specific reverse primers TH359 (for DN5–DH4)

and TH360 (for DN10–DH4), respectively. The vector fragments were ligated to TetR cassette (stuffer fragment of 2101 bp) with SfiI restriction enzyme and the correct double mutant sequences were verified by sequencing. SfiI vector backbones from pEB32x-DARPin vectors parental PelB, DN5, DH4 and DN10, as well as, from pEB32x-Tet double mutant vectors DN5–DH4 and DN10–DH4 were gel extracted and ligated with a combinatorial DARPin library SfiI DNA insert containing serine/tyrosine codon variation (TMY) at specified codon positions (**Figure S1**). The DARPin binary library was purchased as ready linear DNA block from Eurofins Genomics (Ebersberg, Germany), amplified with PCR using added flanking primer sites and digested with SfiI. The ligated library DNA samples were transformed to *E. coli* XL-1 Blue cells with electroporation each yielding >10 000 cfu transformants. All PelB mutant libraries contained < 0.7% vector background colonies, which were calculated from the transformation plates of vector control ligations (no insert) that were prepared in parallel to the library samples.

As DsbA sequence cannot contain a compatible SfiI site for receiving the DARPin binary library SfiI DNA fragment, DsbA-DARPin library was constructed with Gibson assembly. The vector fragment containing DsbA was PCR amplified with primers TH363 and TH364 using pEB32x-DsbA-DARPin anti-GFP vector as the template. The DARPin binary library was amplified with primers TH362 and JLe01as using peB32x-DARPin library (parental PelB) as the template. Gibson reaction was performed according to Gibson 2009 paper [37]. The resulting pEB32x-DsbA-DARPin library was further purified by PCR amplification with primers WO375 and PAKrev, XbaI and HindIII digestion and gel extraction of the correct size fragment covering DsbA-DARPin-p3-CT gene. The insert was ligated to XbaI and HindIII digested pEB32x vector fragment and electroporated to *E. coli* XL-1 Blue cells yielding 1.6 $x10^4$ cfu transformants with 3% vector background colonies that were calculated as above. The library cloning strategy was confirmed by sequencing ten DN10–DH4 (same insert in all PelB libraries) and ten DsbA clones. In both library samples 7/10 clone sequences were according to the library design and 3/10 frameshift clones.

Cloning of pAK400-TrxA and -DARPin periplasmic export constructs

Soluble DARPin anti-GFP periplasmic expression vectors (WT, DN5 A8V, DN5 V3 and DN5-DH4) were constructed by inserting XbaI-PstI fragments from pEB32x-DARPin (contain signal sequence and DARPin gene) into pAK400-DARPin PstI-XbaI backbone and verified by

sequencing. For the TrxA export reporter constructs, TrxA was amplified with primers TH393 and TH394 (Table S1) from the genome of *E. coli* XL-1 Blue cells with Phusion DNA polymerase. To this end, a colony of cells was resuspended in 50 µl H_2O, boiled for 5 min at 98 °C and, 1 µl of thermally lysed cells was added to a 50 µl PCR reaction as template. PCR was cycled for a total of 40 cycles (init. den. at 98 °C for 30 s, den. at 69 °C for 10 s, ext. at 72 °C 30 s and final ext. at 72 °C for 5 min) and the product purified with DNA Clean & Concentrator-5 Kit (Zymo Research, USA). TrxA was cloned with flanking SfiI sites to pAK400 and verified by DNA sequencing. pAK400-TrxA PelB sequence variants (WT, DN5 A8V, DN5 V8A, DN5 V3 and DN5-DH4) were prepared by replacing DARPin gene in pAK400 vectors by TrxA gene with SfiI. The pAK400-DARPin DsbA control used for the western blotting experiment was cloned with Gibson assembly as described above using primers TH419, TH420, TH421 and TH422. $PhoA_{WT}$-TrxA, $PhoA_{GTG}$-TrxA and TrxA without signal sequence were cloned by amplifying TrxA gene form pAK400-TrxA $PelB_{WT}$ with forward primers TH436, TH437 and TH438 and reverse primer pAKrev2 and, cloning the PCR products to pAK400 vector as XbaI-HindIII fragment.

LC-ESI-MS

The purified periplasmic DARPin samples were diluted to 0.1 µg/µl concentration with ultrapure water and 0.2 µg protein was used for LC-ESI-MS. The LC-ESI-MS analyses were performed on a nanoflow HPLC system (Easy-nLC1000, Thermo Fisher Scientific) coupled to the Orbitrap Fusion Lumos Tribrid mass spectrometer (Thermo Fisher Scientific, Bremen, Germany) equipped with a nano-electrospray ionization source. The samples were first loaded into a trapping column (2 cm, 100 µm i.d.) and then separated online in an analytical column (15 cm, 75 µm i.d.). The both columns were in-house packed with ReproSil-Gold 300 C4 3 µm column packing material (Dr. Maisch Gmbh, Germany). The mobile phases consisted of a solvent A (water with 0.1% formic acid) and a solvent B (acetonitrile/water (80:20 (v/v)) with 0.1% formic acid). The online desalting was carried out by loading the samples into the precolumn with 22 µl volume of the solvent A. A 20 min chromatographic method was used to elute proteins: from 12% to 65% of the solvent B in 14 min, following from 65% to 100% of the solvent B in 3 min, and finally 3 min wash at 100% solvent B. A flow rate was 400 nl/min.

MS data was acquired automatically by using Thermo Xcalibur 4.4 software (Thermo Fisher Scientific). A scan range from m/z 420 to m/z 2000 at 7500 orbitrap resolution was used in the MS method. A performance of the instrumentation was checked by analyzing a protein standard mixture (Pierce intact protein standard, Thermo Fisher Scientific) before the sample analyses.

Protein spectra were charge state deconvoluted and average masses and protein abundances were calculated by using BioPharma Finder 2.0 (Thermo Fisher Scientific) software.

Supplementary Figures and Tables

```
    SfiI            D  L  G  K  K  L  L  E  A  A  Z  Z  G  Q  D  D  E  V  R  I  L  M
GGCCCAGCCGGCCATGGCGGATCTGGGTAAAAAACTGCTGGAAGCAGCATMYTMYGGTCAGGATGATGAAGTTCGTATTCTGATG
 A  N  G  A  D  V  N  A  Z  D  Z  Z  G  Z  T  P  L  H  L  A  A  Z  Z  G  H  L  E  I
GCAAATGGTGCAGATGTTAACGCCTMYGACTMYTMYGGTTMYACACCGCTGCATCTGGCAGCATMYTMYGGTCATCTGGAAATTG
V  E  V  L  L  K  H  G  A  D  V  N  A  Z  D  Z  Z  G  Z  T  P  L  H  L  A  A  Z  Z  G
TTGAAGTTCTGCTGAAACATGGTGCCGATGTGAATGCGTMYGATTMYTMYGGTTMYACCCCGCTGCACTTAGCAGCATMYTMYGG
  H  L  E  I  V  E  V  L  L  K  Y  G  A  D  V  N  A  Z  D  Z  Z  G  Z  T  P  L  H  L
CCATTTAGAAATCGTGGAAGTTTTACTGAAATATGGAGCTGATGTTAATGCCTMYGATTMYTMYGGTTMYACGCCTTTACATCTG
 T  A  Z  Z  G  H  L  E  I  V  E  V  L  L  K  H  G  A  D  V  N  A  Q  D  Z  Z  G  Z
ACCGCATMYTMYGGCCACCTGGAAATAGTAGAGGTGCTGCTTAAACACGGTGCGGATGTAAATGCACAGGATTMYTMYGGCTMYA
T  A  F  D  L  A  I  Y  Y  G  N  E  D  I  A  E  V  L  Q  K  A  A        SfiI
CCGCCTTTGATCTGGCAATTTACTACGGCAATGAAGATATCGCAGAAGTTCTGCAAAAAGCAGCGTCGGCCACGGGGGCC
```

Figure S1. SfiI DNA fragment of DARPin library containing TMY codons (Z) at selected positions leading to random binary amino acid diversity (Serine/Tyrosine) in the translated repertoire. Underlined sequences indicate the SfiI-restriction sites used for cloning the insert into mutant PelB signal sequence vectors. The first (D1) and last (A157) residue position of the displayed DARPin molecules are bolded.

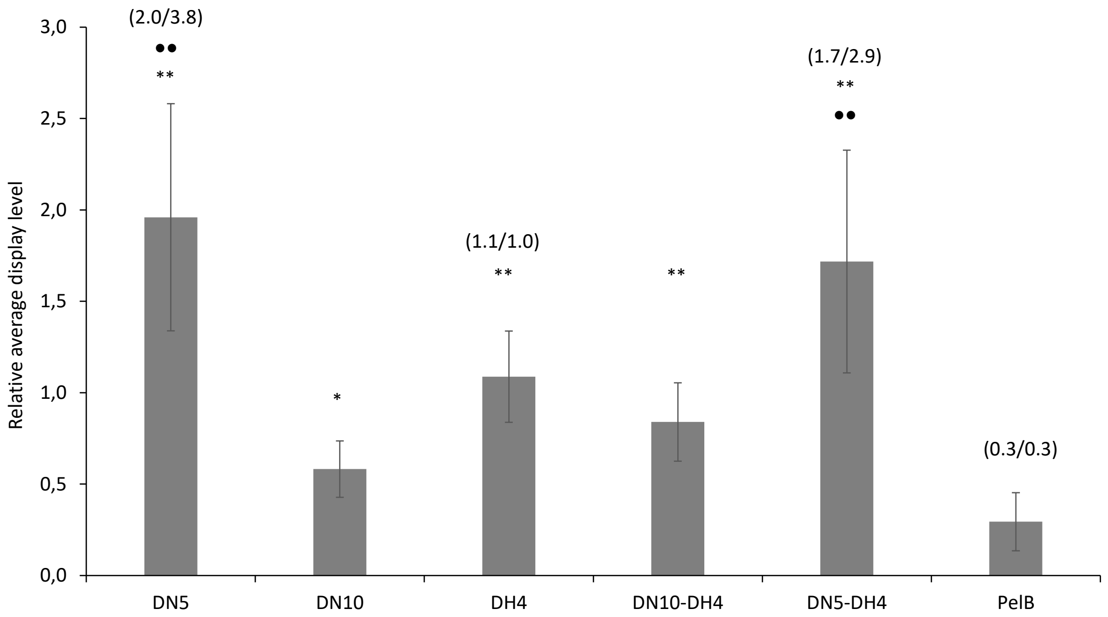

Figure S2. Effect of synonymous codons of PelB signal sequence on average display levels of the DARPin serine/tyrosine binary library with alternative production protocol at 37 °C. Phage production was performed by growing phagemid harbouring XL-1 Blue cells in SB medium with 1 % w/v glucose for superinfection with VCSM13. After infection, the medium was exchanged to SB medium without glucose and phage production propagated overnight at 37 °C. All the average display levels are relative to the display levels of binary library displayed with DsbA signal sequence. The PelB-variant/DsbA ratios for the old (O) and new (N) protocol are indicated over the bars in applicable samples as (O/N). DN10 and DN10-DH4 clones were not studied further in other phage productions as DN5, and derivates thereof, showed higher display efficiency and, therefore, lack the comparative value form the other production method (-). DN5 contains additional A8V mutation in h-region, whereas DN5-DH4 contains only synonymous codon changes. Error bars represent standard deviation of five independent phage productions. Significant differences to the parental PelB and DsbA are shown by asterisk (*) and sphere (●), respectively. Mann-Whitney U, *(●) $p < 0.05$; **(●●) $p < 0.01$.

A. $PelB_{WT}$-TrxA

```
    XbaI              M  K  Y  L  L  P  T  A  A  A  G  L  L  L  L  A  A  Q  P  A  M  A
ATTTCTAGATAACGAGGTCAAATCATGAAATACCTATTGCCTACGGCAGCCGCTGGATTGTTATTACTCGCGGCCCAGCCGGCCATGGCG
 S  D  K  I  I  H  L  T  D  D  S  F  D  T  D  V  L  K  A  D  G  A  I  L  V  D  F  W  A  E
AGCGATAAAATTATTCACCTGACTGACGACAGTTTTGACACGGATGTACTCAAAGCGGACGGGGCGATCCTCGTCGATTTCTGGGCAGAG
 W  C  G  P  C  K  M  I  A  P  I  L  D  E  I  A  D  E  Y  Q  G  K  L  T  V  A  K  L  N  I
TGGTGCGGTCCGTGCAAAATGATCGCCCCGATTCTGGATGAAATCGCTGACGAATATCAGGGCAAACTGACCGTTGCAAAACTGAACATC
 D  Q  N  P  G  T  A  P  K  Y  G  I  R  G  I  P  T  L  L  L  F  K  N  G  E  V  A  A  T  K
GATCAAAACCCTGGCACTGCGCCGAAATATGGCATCCGTGGTATCCCGACTCTGCTGCTGTTCAAAAACGGTGAAGTGGCGGCAACCAAA
 V  G  A  L  S  K  G  Q  L  K  E  F  L  D  A  N  L  A  S  A  T  G  A  D  H  H  H  H  H  *
GTGGGTGCACTGTCTAAAGGTCAGTTGAAAGAGTTCCTCGACGCTAACCTGGCGTCGGCCACGGGGGCCGATCACCATCATCACCATTAG
 HindIII
TAAGCTTGAC
```

B. $PhoA_{GTG}$

```
     XbaI             M K  Q  S  T  I  A  L  A  L  L  P  L  L  F  T  P  V  T  K  A
ATTTCTAGATAACGAGGTCAAATCGTGAAACAAAGCACTATTGCACTGGCACTCTTACCGTTACTGTTTACCCCTGTGACAAAAGCC
```

Figure S3. PelB variant-TrxA constructs used in TrxA export reporter study. The first and last codon of TrxA are bolded. A) The full construct with $PelB_{WT}$ signal sequence (boxed). B) PhoA signal sequence. The boxed sequence contains the signal sequence and is different between pAK400-TrxA constructs. Both native PhoA signal sequence with a start codon of GTG ($PhoA_{WT}$)and a mutant with start codon ATG were tested in parallel in the TrxA export experiment. Underlined sequences indicate the restriction sites used for cloning the inserts $PhoA_{ATG}$-TrxA, $PhoA_{GTG}$-TrxA and the no signal sequence variant, in which the boxed sequence was ATG only.

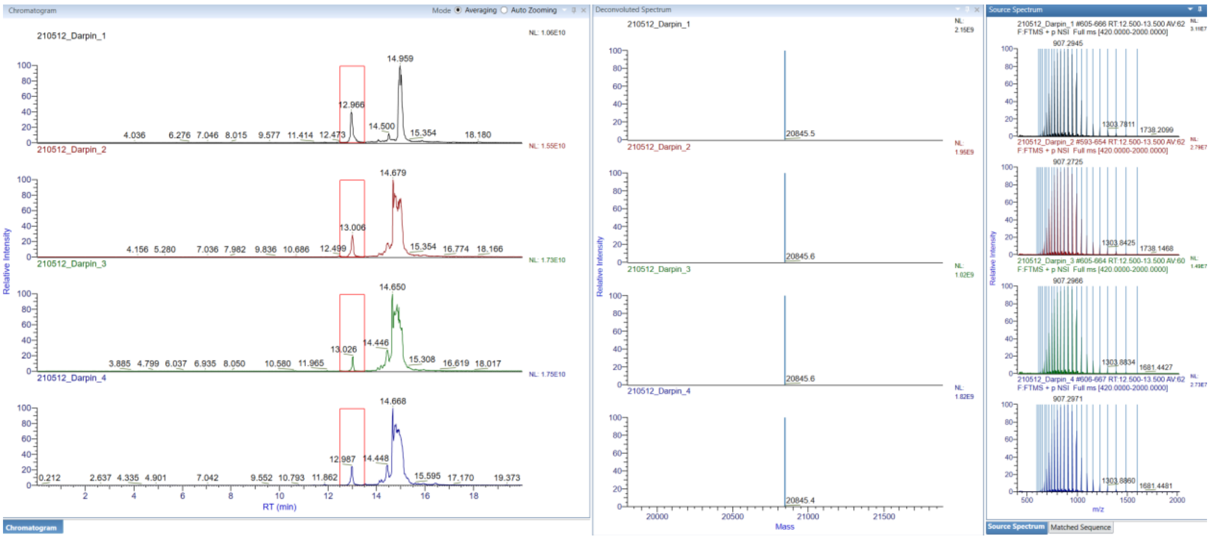

Figure S4. Total ion chromatograms (left pane), deconvoluted average masses (middle pane) and average spectra (right pane) of the samples Darpin-1, Darpin-2, Darpin-3 and Darpin-4 are shown in the figure. Average masses and spectra shown in here were calculated from the retention time range 12.5 – 13.5 min (red rectangle in the left pane). Normalized intensity (NL) of the most abundant peak in each sample is shown in the right corner of the pane.

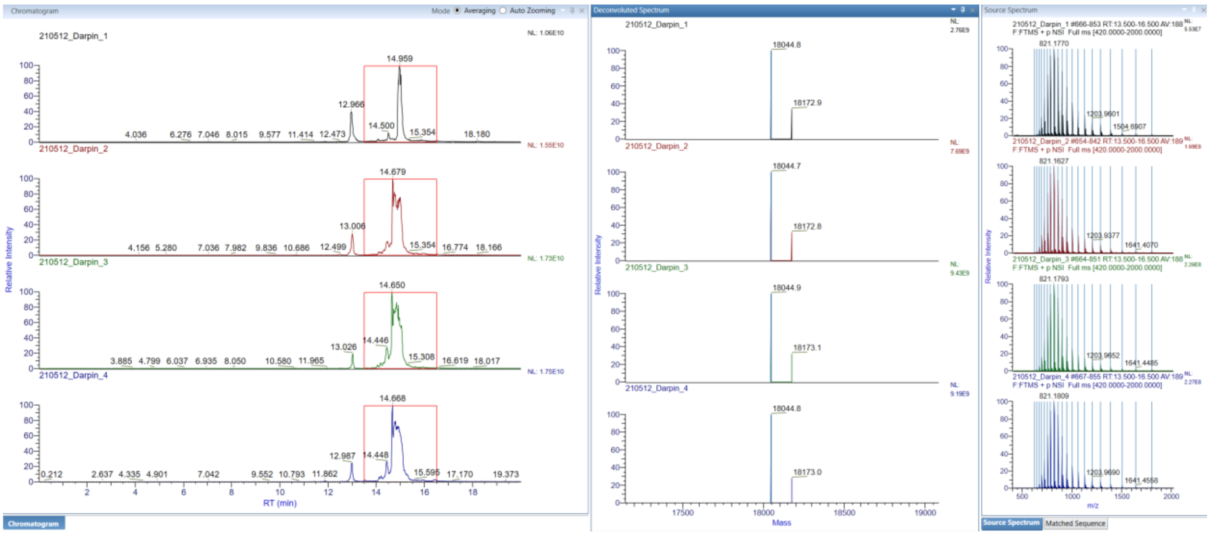

Figure S5. Total ion chromatograms (left pane), deconvoluted average masses (middle pane) and average spectra (right pane) of the samples Darpin-1, Darpin-2, Darpin-3 and Darpin-4 are shown in the figure. Average masses and spectra shown in here were calculated from the retention time range 13.5 – 16.5 min (red rectangle in the left pane). Normalized intensity (NL) of the most abundant peak in each sample is shown in the right corner of the pane.

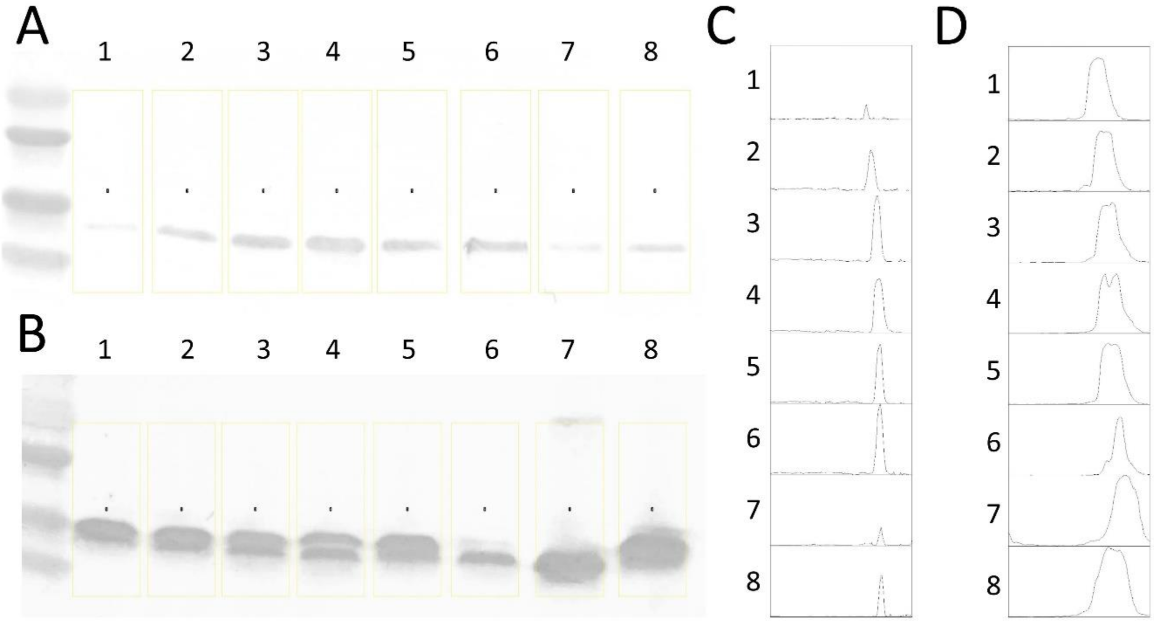

Figure S6. Densitometry of TrxA reporter assay western blots. A) An example of lanes defined with Fiji (ImageJ 1.53c, W. Rasband, NIH, USA) from periplasmic extract western blot. B) An example of lanes defined with Fiji from whole cell western blot. C) The equivalent profile plot of the periplasmic extract samples used for determining the peak areas corresponding to band sizes. D) The equivalent profile plot of the whole cell samples used for determining the peak areas corresponding to band sizes. Quantitated sample set: 1) PelB$_{WT}$; 2) PelB DN5 V8A; 3) PelB DN5 A8V; 4) PelB DN5 V3; 5) DN5-DH4, 6) DsbA, 7) without signal sequence and 8) PhoA.

Table S1. Primers used in the study

Name	DNA sequence 5´-3´
TH359	CATGGCCGGCTGGGCCGCAAGAAGAAGTAGACCAGCTGCTGCGGTGGGGAGGAGATATTTC
TH360	CATGGCCGGCTGGGCCGCAAGAAGAAGTAGACCAGCTGCTGCCGTCGGTAGAAGGTACTTC
TH361	TCCTGCAGAAACTGGGAGCGG
TH362	TAGCGTTTAGCGCATCGGCGGATCTGGGTAAAAAACTGCTG
TH363	TGAAGGTCGTGGCGGTTCTGGTTC
TH364	ATCCGCCGATGCGCTAAACGC
TH393	ATTGGCCCAGCCGGCCATGGCGAGCGATAAAATTATTCACCTGACTGACG
TH394	CTCGGCCCCCGTGGCCGACGCCAGGTTAGCGTCGAGG
TH419	TCGGCCACGGGGGCCGAT
TH420	CGCCGATGCGCTAAACGCTAAAACTAAACCAG
TH421	TAGCGTTTAGCGCATCGGCGAGCGATAAAATTATTCACCTGACTGAC
TH422	TGATCGGCCCCCGTGGCCGACGCCAGGTTAGCGTCGAG
TH436	AATTTCTAGATACGAGGGCAATCATGAAACAAAGCACTATTGCACTGG
TH437	AATTTCTAGATACGAGGGCAATCGTGAAACAAAGCACTATTGCACTGG
TH438	AATTTCTAGATACGAGGGCAATCATGAGCGATAAAATTATTCACCTGACTGACG
PAKrev	CGCCATTTTTCACTTCACAG
JLe01as	ACCAGAACCGCCACGACCTTCAA
WO375	TCACACAGGAAACAGCTATGAC
